# Differences in clinical characteristics and tumor prognosis between primary and secondary conventional pelvic chondrosarcoma

**DOI:** 10.1186/s12885-020-07530-9

**Published:** 2020-11-02

**Authors:** Jie Zang, Wei Guo, Rongli Yang, Xiaodong Tang, Haijie Liang

**Affiliations:** Musculoskeletal Tumor Center, Peking University People’s Hospital, No.11 Xizhimen South Street, Xicheng District, Beijing, 100044 China

**Keywords:** Chondrosarcoma, Primary, Secondary, Pelvis

## Abstract

**Background:**

Chondrosarcoma (CS) most commonly involves the pelvis. This study aimed to analyze differences in clinical characteristics and prognostic factors between primary and secondary conventional pelvic CS, and provide reference for clinical diagnosis and treatment.

**Methods:**

Eighty patients (54 primary cases and 26 secondary cases) with pelvic CS were included in this retrospective study. The tumor site, Enneking stage, soft tissue mass, margin, initial tumor grade, incidence of local recurrence and distant metastasis were evaluated. Kaplan-Meier method was used to calculate the overall survival rate. X^2^ test and log-rank test were used for univariate analysis, and Cox test was used in multivariate analysis.

**Results:**

The average age of patients with secondary CS was significantly younger than that of patients with primary CS (*P* < 0.001). The soft tissue mass of patients with secondary CS was significantly larger than that of patients with primary CS (*P* = 0.002). There was a significant difference in initial tumor pathologic grade between the two groups (*P* = 0.002). No statistically significant difference was observed in the local recurrence rate between the two groups. The median recurrence time of patients with primary CS after the first treatment was significantly shorter than that of patients with secondary CS (*P* < 0.001). The overall survival rate of patients with secondary CS was much higher than that of patients with primary CS (*P* = 0.003). Cox regression analysis showed that the initial tumor grade was an independent factor in the overall survival rate of patients with CS.

**Conclusion:**

There were significant differences in age, soft tissue mass, initial tumor grade, and overall survival rate between the two groups. The overall survival rate of pelvic CS was related to the initial tumor grade of CS.

## Background

Chondrosarcoma (CS) is a malignant cartilaginous matrix-producing bone tumor [1]. Its incidence is only next to that of osteosarcoma, accounting for 17–24% of malignant bone tumors. The pelvis is involved in about 22–39% of CS patients [2, 3]. CS has many subtypes, among which conventional CS is the most common, accounting for more than 90% of CS cases [4]. According to pathological classification, conventional CS can be divided into grade I to III. Primary CS occurs in the bone without a pre-existing lesion. If the tumor develops from a pre-existing benign tumor such as osteochondroma or enchondroma, it is called secondary CS. From its location, it can further be divided into the central type and peripheral type. The former occurs in the marrow cavity, while the latter occurs in the bone surface, generally from the cartilage cap of an osteochondroma [5]. Primary CS is always central and has early onset characteristics, a high degree of malignancy, rapid development, and a poor prognosis [6]. It is mainly found in adults and older people, and the incidence rate in males is higher than that in females [7–10]. Secondary CS is rare, and the literature on secondary CS is relatively limited. It could be central or peripheral, and has the characteristics of late onset, slow development, and a good prognosis. It needs clinical, imaging, anatomical, and pathological analysis to make a correct diagnosis and histological classification. In a retrospective analysis of 107 patients with secondary CS, Adel et al. found that patients with secondary CS were 1–20 years younger than those with primary CS. The local recurrence rates of patients with secondary CS were 15.9 and 17.5% at 5 and 10 years. The 5-year and 10-year mortality rates of patients receiving the first treatment were 1.6 and 4.8%, respectively [11]. However, a direct comparison between primary and secondary CS in clinical characteristics has not been reported.

CS is not sensitive to radiotherapy and chemotherapy, and surgical treatment is the primary treatment [1]. Previous studies have shown that *en bloc* resection with an adequate surgical margin is necessary to achieve local control in any pathological tumor grades [7, 12]. However, pelvic CS is large, complex in local structure, and adjacent to blood vessels and nerve bundles, making it very difficult to operate [8, 13, 14]. It has been reported that the prognostic factors include tumor grade, tumor size, surgical margin, and tumor type (primary or secondary tumors) [7–9, 15]. However, these reports contain other pathological subtypes, and there is no direct comparison of tumor prognosis between primary and secondary CS.

In this study, we retrospectively analyzed 80 patients with pelvic CS to explore the differences in clinical characteristics, local control rate, overall survival rate, and prognostic risk factors between primary and secondary conventional pelvic CS.

## Methods

**Fig. 1:**
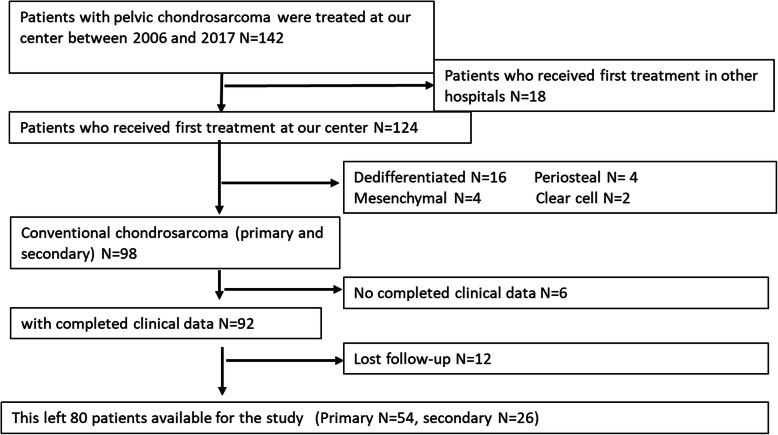
The study flowchart

A total of 142 cases of pathologically confirmed pelvic CS in our hospital from 2006 to 2018 were collected. Among them, 18 patients were excluded because the first treatment was received in other hospitals. Sixteen cases of dedifferentiated subtype, 4 cases of periosteal type, 4 cases of mesenchymal type and 2 cases of clear cell type were excluded. Thereafter, 6 patients did not have complete clinical data, and 12 patients were lost to follow-up. Finally, 80 cases of conventional CS were included in the study, including 54 cases of primary CS and 26 cases of secondary CS (Fig. 1). All patients with secondary CS received a previous pathological diagnosis of benign chondropathy, and were followed up by our hospital before the diagnosis of secondary CS. In the secondary CS group, CS originated from enchondromas in 4 cases, osteochondromas in 8 cases, Ollier disease in 4 cases, and multiple osteochondromas in 10 cases.

According to the pathological results, combined with clinical and imaging data, the tumor’s surgical stage was determined according to the Enneking malignant tumor classification [16] to determine the optimal treatment plan. The histological classification was made according to the system proposed by Evans et al. [17]. According to the Enneking classification [18], the surgical margin was determined, which included intralesional, marginal, and wide margins.

Within two years after the operation, the patients were followed up every three months by visit to the outpatient department. The X-ray films of the operation site were rechecked, and CT or MRI was performed as required. Chest X-ray or CT scan was used to determine whether there was lung metastasis, and whole-body bone scan was used to determine whether there was bone metastasis. If there was no sign of progression, the patients were followed up every six months after two years and yearly after five years. The follow-up records included local recurrence and distant metastasis. If the patient died, the time of death was confirmed by telephone follow-up. The follow-up time was defined from the beginning of diagnosis to the end of death or statistics.

### Statistical analysis

IBM SPSS statistics 24.0 software was used for data collection and statistical analysis. Pearson χ 2 test was used to test the correlation between two classification variables. If the frequency was < 5, Fisher exact probability method was used. The 5-year and 10-year overall survival estimated by Kaplan-Meier method was defined as the time from the beginning of diagnosis to the end of disease-related death and was censored at the date of the latest follow-up or death due to other causes. Cox regression model was used to analyze the survival time. When these variables were included in Cox regression model for multivariate analysis, the forward method based on maximum likelihood estimation was used for independent variable selection. *P* < 0.05 indicated statistically significant difference.

## Results

### Clinical characteristics of pelvic CS

The majority of patients with primary pelvic CS were over 40 years old (79.6%) while only 19.2% patients with secondary pelvic CS were over 40 years old (*P* < 0.001). Among both the primary and secondary CS groups, the periacetabulum and ischium-pubis was the most common tumor site, which was 46 and 34.6%, respectively (*P* = 0.023). The average diameter of primary soft tissue mass was 6.6 ± 4.3 cm, much shorter than that of secondary soft tissue mass (10.6 ± 4.5 cm) (*P* = 0.002). Moreover, 53.7% of primary CS patients had a tumor diameter < 8 cm, while only 30.8% of secondary CS patients had a tumor diameter < 8 cm. In the primary CS group, 4 cases of patients had lung metastases and were classified as stage III. The remaining 50 cases had localized diseases. In the secondary CS group, no case had lung metastasis and all the 26 cases had localized diseases. In the secondary CS patients, 26.9% had grade I CS initially versus 7.4% in the primary CS patients. In addition, 22.2% of primary CS patients had grade III CS while no secondary CS patients had grade III CS. There was no significant difference in Enneking stage and margin between the two groups (Table 1).

**Table 1:** Comparison of primary and secondary chondrosarcoma of pelvis in terms of clinical characteristics

Variable		Primary	Secondary	*P*
Gender	Male	29 (53.7%)	18 (69.2%)	0.23
	Female	25 (46.3%)	8 (30.8%)	
Age	Mean ± SD (years)	48.5 ± 12.8	32.2 ± 8.8	0.000
	< 40 (years)	11 (20.4%)	21 (80.8%)	0.000
	≥40 (years)	43 (79.6%)	5 (19.2%)	
Tumor Site	I	5 (9.3%)	7 (26.9%)	0.023
I:illium	I + P	6 (11.1)	2 (7.7%)	
P:periacetabulum	I + P + I-P	10 (18.5%)	1 (3.8%)	
I-P:ischium-pubis	I + P + I-P + S	2 (3.7%)	0	
F:femoral	I + P + I-P + F	1 (1.9%)	0	
S:sacrum	I + P + S	1 (1.9%)	0	
	I + S	1 (1.9%)	0	
	P	3 (5.6%)	1 (3.8%)	
	P + I-P	25 (46%)	9 (34.6%)	
	P + I-P + F	0	1 (3.8%)	
	I + P	0	4 (15.4%)	
	I + P + F	0	1 (3.8%)	
Enneking Stage	IA	1 (1.9%)	2 (7.7%)	0.439
	IB	3 (5.6%)	2 (7.7%)	
	IIA	6 (11.1%)	1 (3.8%)	
	IIB	40 (74.1%)	21 (80.8%)	
	III	4 (7.4%)	0	
Soft Tissue Mass	Mean ± SD (cm)	6.6 ± 4.3	10.6 ± 4.5	0.002
	<8 cm	29 (53.7%)	8 (30.8%)	0.016
	≥8 cm	25 (46.3%)	18 (69.2%)	
Margin	Wide	22 (40.7%)	10 (38.5%)	0.809
	Marginal	19 (35.2%)	8 (30.8%)	
	Intralesional	13 (24.3%)	8 (30.8%)	
Initial Tumor Grade	I	4 (7.4%)	7 (26.9%)	0.002
	II	38 (70.4%)	19 (73.1%)	
	III	12 (22.2%)	0	

### Follow-up results of pelvic CS

The follow-up results showed that the disease-free survival rate and overall survival rate were 50 and 55.6% for patients with primary CS, and 65.4 and 84.6% for patients with secondary CS, respectively. The difference in survival rate between the two groups was significant (*P* = 0.016). The mortality rate of patients with primary CS was 44.4%. Among them, 2 cases died during the perioperative period (1 case died of cerebral hemorrhage, and 1 case died of septic shock), 2 cases died of cerebral hemorrhage, 1 case died of myocardial infarction, and 1 case died of pulmonary embolism. Twelve cases died of lung metastasis, 4 cases died of cachexia and 2 cases died of brain metastasis. The mortality rate of secondary CS patients was only 15.4%, among whom 2 died of cachexia, and 2 died of lung metastasis. The mean follow-up duration was 40.0 ± 28.2 months for primary CS patients and 68.8 ± 38.6 months for secondary CS patients (Table 2).

**Table 2:** Comparison of postoperative follow-up results of primary and secondary pelvic chondrosarcoma

Variable	Primary	Secondary	*P*
Alive without disease	27 (50%)	17 (65.4%)	0.016
Alive with disease	3 (5.6%)	5 (19.2%)	
Death	24 (44.4%)	4 (15.4%)	
Postoperative hemorrhage (perioperative period)	1 (1.9%)	0	
Septic shock (perioperative)	1 (1.9%)	0	
Cerebral hemorrhage	2 (3.7%)	0	
Myocardial infarction	1 (1.9%)	0	
pulmonary embolism	1 (1.9%)	0	
Pulmonary metastasis	12 (22.2%)	2 (7.7%)	
Cachexia	4 (7.4%)	2 (7.7%)	
Brain metastases	2 (3.7%)	0	
Follow-up time			
Mean ± SD (Months)	40 ± 28.2	68.8 ± 38.6	0.002

### Local recurrence of pelvic CS

The diagnosis of local recurrence includes CT, MRI, PET-CT and biopsy. In this study, 20 patients (37.0%) with primary pelvic CS and 9 patients (34.6%) with secondary pelvic CS had local recurrence, and there was no statistically significant difference between the two groups. Surgical pathological examination revealed that of the 20 patients who relapsed in the primary CS group, 1 was grade I, 14 were grade II, and 5 were grade III. Nine patients relapsed in the secondary CS group, 3 had grade I pathology after the initial operation, and 6 had grade II pathology. The median recurrence time of primary CS patients after the first treatment was 14 months, which was significantly shorter than that of secondary patients (*P* < 0.001) (Table 3).

**Table 3:** Comparison of primary and secondary chondrosarcoma of pelvis in terms of local recurrence

Variable		Primary	Secondary	*P*
Local recurrence,n(%)	No	34 (63.0)	17 (65.4)	0.436
	Yes	20 (37.0)	9 (34.6)	
Recurrence time after first treatment (months)	Median (IQR)	14.0 (8.0–21.8)	38.5 (25.0–59.5)	< 0.001
Pathological progression after recurrence^a^,n(%)	No	7 (58.3)	5 (55.6)	1.000
	Yes	5 (41.7)	4 (44.4)	

^a^Received second surgical treatment: Primary *N* = 12; secondary *N* = 9

*IQR* interquartile range

### Prognosis of pelvic CS

**Fig. 2:**
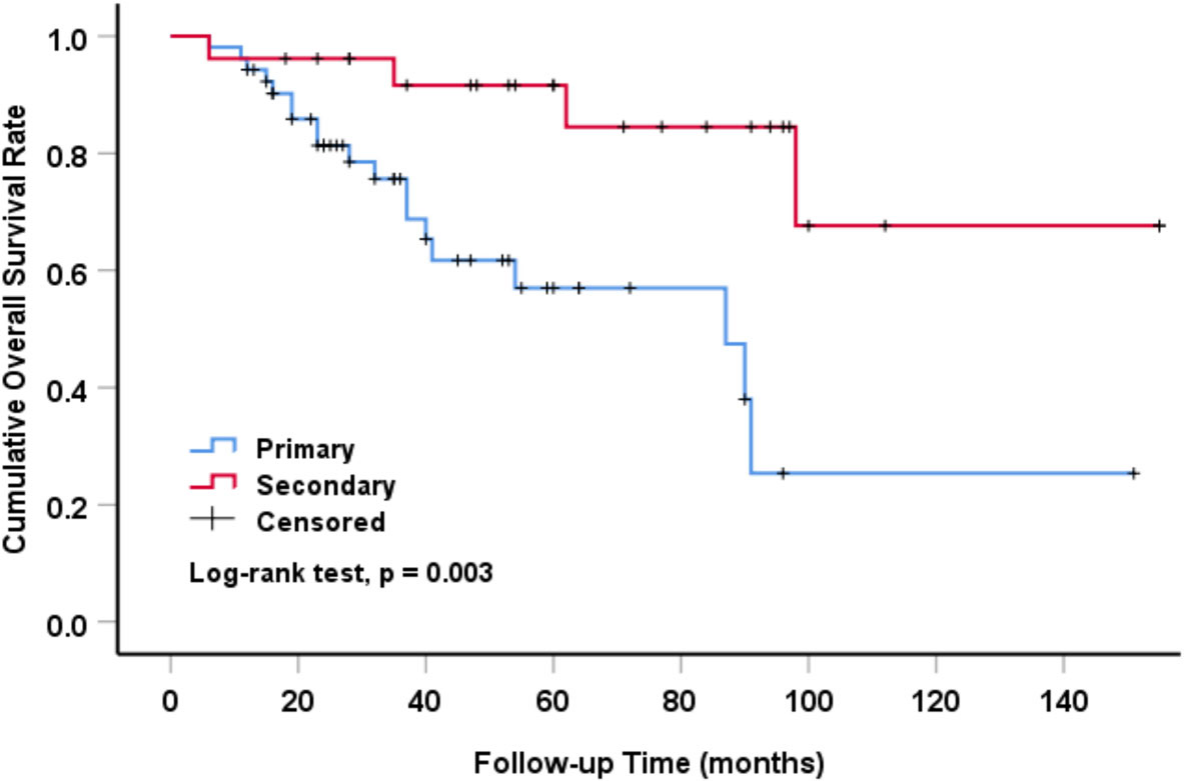
Overall survival of primary and secondary pelvic CS

**Fig. 3:**
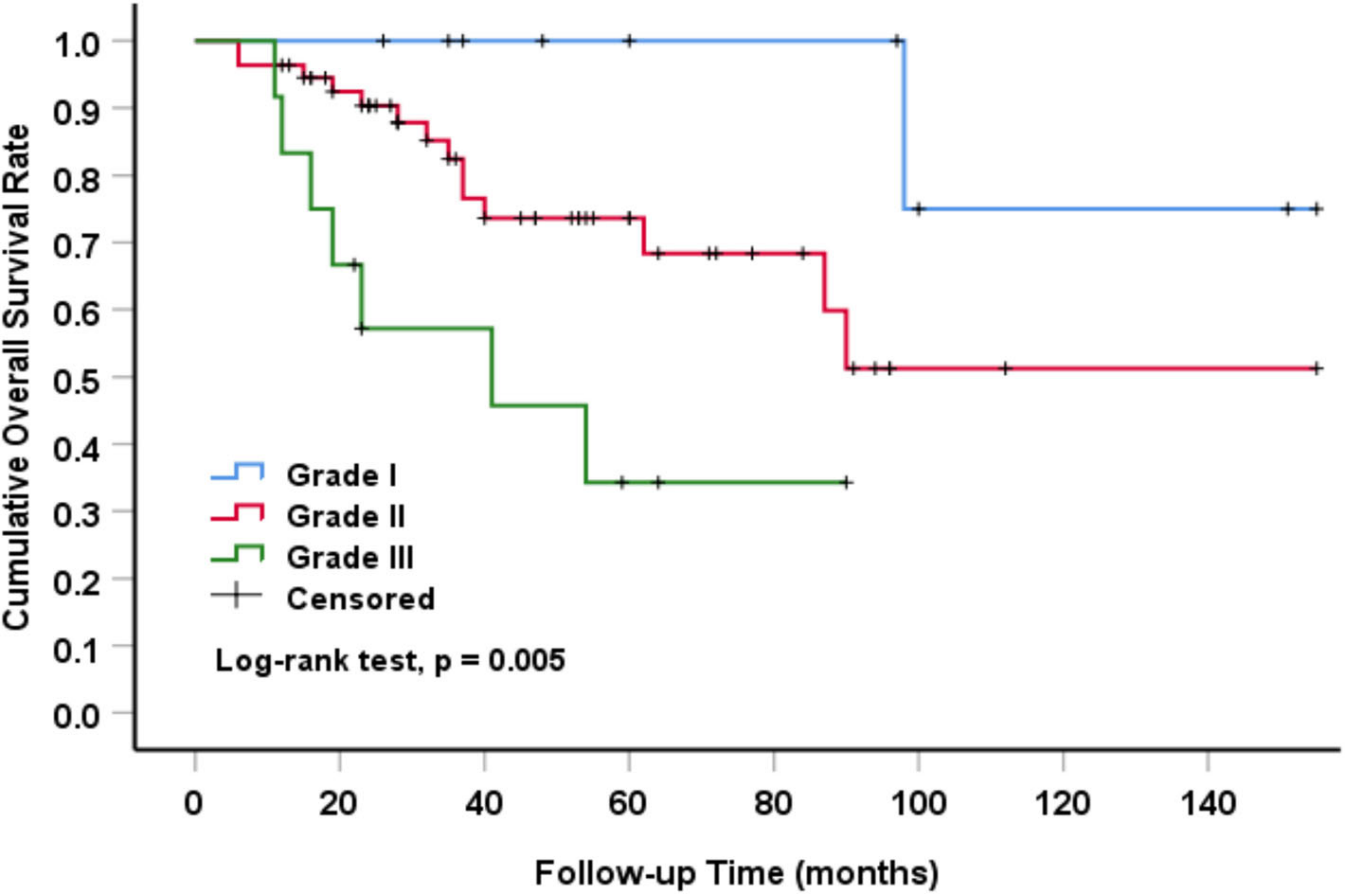
Effect of initial tumor classification on survival of patients with pelvic CS

The overall survival rate estimated by Kaplan-Meier method of primary and secondary pelvic CS patients was significantly different (*P* = 0.003) (Fig. 2). The 5-year and 10-year survival rates of primary CS patients were 57 and 25.3%, respectively. The 5-year and 10-year overall survival rates of secondary CS patients were much higher than those of primary patients, which were 91.6 and 67.6%, respectively. The overall survival time of patients with pelvic CS was analyzed by univariate analysis, and the variables included initial tumor grade. The survival rate was 100% in grade I, 73.6% in grade II and 34.3% in grade III (*P* = 0.005) (Fig. 3). Further multivariate analysis showed that initial tumor grade was an independent prognostic factor. The higher the grade was, the lower the overall survival rate was (Table 4).

**Table 4:** Multivariable analysis designed to analyze independent factors associated with overall survival

Viable	HR	95% CI	*P*
Initial Tumor Grade			0.005
Grade II vs Grade I	14.837	1.110–198.286	0.041
Grade III vs Grade I	161.445	5.954–4377.516	0.003

## Discussion

The treatment of pelvic CS is a major challenge for surgeons. In this study, we compared primary and secondary conventional pelvic CS patients treated at our center in the past 10 years in terms of clinical characteristics, local recurrence, and overall survival. We also determined the prognostic factor associated with overall survival of patients with CS treated with surgery.

In comparing clinical characteristics, we found significant differences in age of onset, the size of soft tissue mass, and pathological tumor grade between the primary and secondary CS group. In this study, the mean age of primary pelvic CS patients was 48.5 years, and the mean age of secondary pelvic CS patients was 32.2 years. The age in the primary CS group is significant older than that for the secondary CS group. This finding is consistent with previous studies [10]. In this study, we also found that patients with pathological grade II accounted for most patients in the primary and secondary CS groups. Still, no case had pathological grade III secondary CS, which may be related to the small number of cases. The pathological grade of secondary CS patients may be lower than that of primary CS patients. In future work, we will continue to observe whether this conclusion is correct.

In this study, the volume of tumor mass in the secondary CS group was larger than that in the primary CS group, which is similar to the results of Ozaki et al. [19]. Our study also found that tumors secondary to osteochondroma and multiple osteochondromatosis accounted for 70% (18/26) of malignant changes in the pelvis. Of these patients, tumors tend to grow out of bone and form large masses, but they do not destroy the bone cortex. When the tumor grows into the pelvic cavity, it is not easy to show symptoms in the early stage, so the tumor volume is often larger at the time of diagnosis.

According to the literature, the local recurrence rate of pelvic CS is 18–45% [7–9, 15, 20–23]. In this study, the local recurrence rate of the primary and secondary CS group were 37.0 and 34.6%, respectively. There was no significant difference in the recurrence rate between the two groups, but there was a difference in recurrence time. Among the recurrent cases, grade I accounted for 5% of the primary CS group and 33.3% of the secondary CS group. Grade I CS has low malignancy and a slow growth rate, which is the cause of late recurrence in the secondary CS group.

In this study, the prognosis of patients in the secondary CS group was significantly better than that in the primary group, with a 5-year survival rate of 91.6 and 57% (*P* = 0.003), respectively, which is consistent with the results of Ozaki et al. [19]. Although it has been reported that the prognosis of CS is related to surgical resection margin [15, 20, 21], tumor size [20], age [22], local recurrence [22], surgical type [9], tumor stage [15], and tumor location [9, 21]. However, in this study, we conducted a multivariate analysis of all patients with pelvic CS and found that pathological tumor grade, but not tumor type (primary and secondary), was an independent risk factor for prognosis (Table 5). This may be due to the small sample size of this study. In different grades, the prognosis of grade I pelvic CS is the best. The proportion of grade I pelvic CS in the secondary CS group was significantly higher than that in the primary CS group. Therefore, the prognosis of the secondary CS group is significantly better than that of the primary CS group.

**Table 5:** Comparison of literature on pelvic chondrosarcoma

Author	Years of study	Number of patient	Patholy subtype	Pathology grade	Followup (year)	Local recurrence(%)	Surgical margin	Overall survival	Risk factors for OS
Bus et al. [20]	1985 to 2013	162	Conventional primary central	1(*n* = 30),2(*n* = 93),3(*n* = 39)	12.6 (median)	38%	Wide 51%;marginal 26%;intralesional 23%	59%	Tumor grade,resection margins,tumor size,soft-tissue infiltration
Stihsen et al. [22]	1967 to 2012	58	conventional (*n* = 46),dedifferentialted (n = 12)	1(*n* = 11),2(*n* = 22),3(*n* = 13),dedifferentiated (*n* = 12)	13 (mean)	19%	Wide 73%, marginal 17%, intralesional 10%	55% (5-yr)	Tumor grade, age, local recurrence
Donati et al. [9]	1971 to 1999	124	Conventional	1 (n = 1),2(*n* = 44),3(*n* = 18)	NA	18%	Wide 65%, marginal+intralesional 35%	92% (5-yr)	Tumor grade, tumor location (central/peripheral),type of operation (limb-sparing resection/hemipelvectomy),local recurrence
Mavrogenis et al. [7]	1975 to 2008	215	Conventional (*n* = 119),secondary peripheral (*n* = 85), not specified (*n* = 9),periosteal (n = 2)	1(*n* = 58),2(*n* = 105),3(*n* = 20),dedifferentiated (*n* = 32)	8.6 (mean)	30%	Wide 70%;marginal 13%;intralesional 17%	83%(5-yr)	Tumor grade
Ozaki et al. [19]	1970 to 1993	31	Primary (*n* = 23), secondary (n = 8)	NA	5 (median)	45%	Wide 26%;marginal 23%;intralesional 51%	67.7%(5-yr)	Tumor type
Deloin et al. [21]	1968 to 2003	59	Primary (*n* = 47), secondary (n = 12)/conventional(*n* = 52),dedifferentiated (*n* = 7)	1(n = 11),2(*n* = 36),3(n = 5),dedifferentiated (*n* = 7)	7.8 (mean)	31%	Wide 78%;marginal 3%;intralesional 19%	66%(5-yr)	Resection margin, tumor grade, acetabular involvement
Wirbel et al. [15]	1978 to 1998	51	NA	NA	6.2 (mean)	20.40%	Wide 53%;marginal 31%;intralesional 16%	58%	Tumor stage, resection margin
Sheth et al. [23]	1970 to 1992	67	Conventional (*n* = 54),dedifferentialted (n = 13)	1(*n* = 19),2(n = 18),3(*n* = 17),dedifferentiated (*n* = 13)	9.6(median)	28%	Negative 57%, positive 43%	52%	Tumor grade
Guo et al. [24]	1998 to 2007	45	Conventional (*n* = 32),dedifferentialted (n = 9), mesenchymal(n = 4)	NA	3.1 (median)	22.20%	Wide 77%;marginal 7%;intralesional 16%	62.6% (5-yr)	NA
Pring et al. [8]	1975 to 1996	64	Conventional (*n* = 57), dedifferentiated (n = 7)/primary (*n* = 49), secondary (*n* = 15)	1(*n* = 33),2(n = 23),3(n = 1),dedifferentiated (n = 7)	11.7 (median)	19%	Wide 63%;marginal 20%;intralesional 17%	82% (5-yr)	Tumor grade
Current study	2006 to 2018	80	Conventional/ primary (n = 54), secondary (*n* = 26)	I (n = 11),II(n = 57),III(n = 12)	3.3 (median)	37.2%	Wide 63%;marginal 20%;intralesional 17%	67.7%(5-yr)	Tumor grade

There are some limitations in our research. First, the follow-up time is relatively short. CS is characterized by slow growth and late recurrence. Therefore, it is not easy to find a small recurrent tumor due to the influence of surgery and prosthesis. We need to further extend the follow-up duration to observe the difference in recurrence between the two groups. Second, because the samples are difficult to obtain, the sample size difference between the two groups is large, and the total sample size is small. We will conduct studies with larger sample sizes in the future.

## Conclusion

In conclusion, there are differences in age, tumor size, and pathological grade between primary and secondary pelvic CS. There was no difference in local recurrence rate between the two groups. The overall survival rate of patients with secondary pelvic CS was higher than that of patients with primary pelvic CS. The overall survival of pelvic CS was correlated with tumor pathological grade.

## Data Availability

The datasets generated and analyzed during the current study are available from the corresponding author on reasonable request.

## Abbreviations

CS: Chondrosarcoma
LR: Local recurrence
CT: Computed tomography
MRI: Magnetic resonance imaging
PET: Positron emission tomography
